# Supplementary material for: Genome-wide CRISPR Screens in T Helper Cells Reveal Pervasive Crosstalk between Activation and Differentiation
Source: Cell. 2019 Feb 7;176(4):882–896.e18. doi: 10.1016/j.cell.2018.11.044 (PMC6370901; doi:10.1016/j.cell.2018.11.044)
Supplement: Data S2. Processed Data from All the Steps of the Analysis, Related to Figure 1 [file mmc2.zip › supplemental data/motif analysis/Pparg_homer/homerResults/motif3.similar.html]

motif3

## Information for motif3

G
T
A
C
C
T
G
A
A
G
T
C
C
G
A
T
C
A
G
T
A
T
G
C
T
A
G
C
A
C
G
T
A
T
C
G
G
A
C
T
  
Reverse Opposite:  

C
T
G
A
T
A
G
C
T
G
C
A
A
T
C
G
T
A
C
G
G
T
C
A
G
C
T
A
T
C
A
G
G
A
C
T
C
A
T
G
  

|  |  |
| --- | --- |
| p-value: | 1e-606 |
| log p-value: | -1.397e+03 |
| Information Content per bp: | 1.568 |
| Number of Target Sequences with motif | 2593.0 |
| Percentage of Target Sequences with motif | 20.71% |
| Number of Background Sequences with motif | 2299.8 |
| Percentage of Background Sequences with motif | 6.42% |
| Average Position of motif in Targets | 100.0 +/- 52.1bp |
| Average Position of motif in Background | 98.5 +/- 62.6bp |
| Strand Bias (log2 ratio + to - strand density) | 0.0 |
| Multiplicity (# of sites on avg that occur together) | 1.11 |
| Motif File: | file (matrix) reverse opposite |

### Similar de novo motifs found

|  |  |  |  |  |  |  |  |
| --- | --- | --- | --- | --- | --- | --- | --- |
| Rank | Match Score | Redundant Motif | P-value | log P-value | % of Targets | % of Background | Motif file |
| 1 | 0.959 | G A T C T C G A A G T C G C A T A C G T A G T C A G T C C G A T A T C G G A C T A G C T C A G T | 1e-537 | -1238.122321 | 22.01% | 7.76% | motif file (matrix) |
| 2 | 0.911 | A G T C C T G A A G T C C G A T A C G T A G T C A G T C A G C T | 1e-365 | -842.138823 | 16.90% | 6.31% | motif file (matrix) |
| 3 | 0.870 | C T G A A G T C G T C A A C T G A C T G C G T A C G A T C T A G | 1e-359 | -828.547891 | 23.41% | 10.69% | motif file (matrix) |
| 4 | 0.911 | T C G A A T G C A C G T A C G T A G T C A G T C A G C T A T C G | 1e-272 | -626.972181 | 36.14% | 22.23% | motif file (matrix) |
| 5 | 0.722 | C G T A T A C G T A C G C T G A G C T A T C G A A G T C A G T C C T A G A T G C | 1e-214 | -492.856478 | 19.31% | 10.00% | motif file (matrix) |
